# Ultrasound-Assisted Diagnosis of an Intraosseous Foreign Body in a Disc Golf-Related Injury: A Case Study

**DOI:** 10.7759/cureus.110610

**Published:** 2026-06-10

**Authors:** Hector M Rivera-Melo, Simone Jordan, Eric T Stefanowicz

**Affiliations:** 1 Diagnostic Imaging, Southern California University of Health Sciences, Whittier, USA

**Keywords:** diagnostic musculoskeletal ultrasound, disc golf, foreign bodies, metacarpal osteomyelitis, musculoskeletal radiology, orthopedic sports medicine, osteomyelitis

## Abstract

This case describes the imaging findings and clinical details of an adult man who sustained an injury to the third metacarpal and how the use of diagnostic ultrasound was used to assist in the diagnosis of an intraosseous foreign body.

A 31-year-old man presented to an urgent care with redness, swelling, and pain following a history of trauma and laceration to the dorsum of his right hand 24 hours ago. After one week of moderate improvement with oral and intramuscular antibiotic therapy, the plateaued clinical picture prompted imaging with diagnostic ultrasound, which revealed a retained foreign body within the head of the third metacarpal. A non-contrast MRI confirmed the presence of a retained foreign body and demonstrated findings concerning for osteomyelitis. Two days later, the foreign body was removed surgically without complication, and the patient was able to return to normal activities with modifications less than a week after surgery.

## Introduction

Foreign bodies can be introduced into the body through various means, leading to a range of medical emergencies depending on the nature and location of the object. Foreign bodies can be ingested items, such as coins or buttons, or penetrative objects, such as bullets, needles, or glass shards, and can pose various complications depending on their size, material, and location within the body. Penetrating hand injuries are common presentations in urgent care, emergency, orthopedic, and sports medicine settings [1]. Retained foreign bodies may be missed despite wound exploration, irrigation, and initial radiography, particularly when the foreign material is organic and radiolucent [1]. Both timely and correct identification of foreign bodies is important as their presence can lead to significant health risks, ranging from tissue damage to more severe complications, such as vascular injury and sepsis. Accurate assessments are important to prevent serious infections, such as osteomyelitis.

Direct inoculation into the skin is a common cause of musculoskeletal infections [2]. Mechanisms of direct inoculation commonly include open fractures, surgical intervention, and penetrating foreign bodies. Foreign bodies may undergo capsule formation and may result in a painful granulomatous tissue reaction (erythema, edema) [2]. Osteomyelitis is an inflammatory process of bone, which may occur as a result of a foreign body that encounters bone marrow. Most often, the causative agent is bacterial, specifically Staphylococci, of which *Staphylococcus aureus* is most common [3]. Risk factors for developing osteomyelitis include immunosuppression, systemic inflammatory diseases, and advanced age [2]. 

Imaging can play a critical role in the initial detection and safe removal of these objects. The precise localization provided by these imaging techniques aids in planning surgical procedures, ensuring that interventions are effective and as minimally traumatic as possible [4]. Radiologists can use X-rays, computed tomography (CT) scans, magnetic resonance imaging (MRI), and ultrasound to locate and identify foreign bodies. Each modality offers its own unique advantages: X-rays are a cost-effective method of detecting metallic objects; CT scans provide cross-sectional images and superior osseous detail; MRI can identify numerous types of materials and indicate the presence of soft tissue injuries or features of local infection; and ultrasound can identify non-radiopaque bodies such as wood or plastic and provide a real-time imaging perspective [4]. 

Plain radiography is typically the first imaging test for suspected foreign bodies because it can be obtained quickly and is useful for radiopaque materials such as metal or glass [5]. However, wood may be difficult to detect on radiographs because its density can approximate surrounding soft tissues. When clinical suspicion persists after negative or equivocal radiographs, ultrasound is commonly recommended as a next diagnostic step for radiolucent foreign bodies, with CT or MRI reserved for persistent diagnostic uncertainty, deeper structures, or concern for complications [1,5]. Ultrasound evaluation of intraosseous structures is typically limited by the high acoustic impedance of cortical bone, and few reports describe its use in this context. However, in cases of cortical disruption, ultrasound may permit visualization of intramedullary foreign bodies [6].

Imaging findings of osteomyelitis are often consistent regardless of hematogenous versus contiguous spread. Conventional radiographic findings may be occult for the first two weeks following onset, which is dependent on the rate of bone demineralization [7]. Findings may include osteolysis, periosteal reaction, and sequestrum formation [2]. Failure of timely treatment may result in the spread of infection to soft tissues, adjacent joints, abscess formation, pathologic fracture, or systemic infection [8]. Treatment often includes debridement of necrotic tissues, containment of infection, and prolonged antibiotics [8]. Chronic osteomyelitis may result from contiguous soft tissue infection, which is increasing in incidence due to the increasing incidence of diabetes and peripheral vascular disease [9].

## Case presentation

A 31-year-old, Hispanic man with a 15-year history of type 1 diabetes mellitus, managed with an insulin pump and continuous glucose monitor, sustained a penetrating injury to the dorsum of his right hand while competing in a disc golf tournament. During a backhand throw from within a wooded area, the dorsum of the hand struck a tree branch. The patient removed a large wooden splinter from the wound and noticed immediate substantial bleeding. He applied a makeshift bandage (Figure 1A) and completed the round with mild pain. During a one-hour break between rounds, he cleaned and dressed the wound using a liquid bandage and standard adhesive dressing. He then completed a second round of 18 holes. He noted mild pain while gripping and during high-velocity throws. Several hours later, swelling increased, and he became unable to make a full fist without pain. On the following day, swelling had not significantly improved despite icing, and erythema was visible around the injury. The patient marked the border of erythema (Figure 1B). Several hours later, the erythema appeared to be spreading, and swelling worsened (Figure 1C), prompting urgent care evaluation. Upon arrival at urgent care, the wound was lavaged and inspected for foreign bodies. None were found. He was then administered an intramuscular antibiotic injection (Bactrim) and a 10-day course of oral antibiotics (Augmentin).

**Figure 1:**
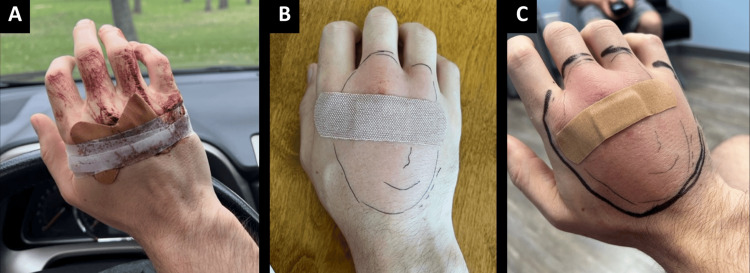
Images of the hand with progressive swelling and erythema. Images of self-bandaged hand shortly after initial injury, (A) approximately 24 hours after the injury demonstrating swelling and erythema with a line drawn around the erythema (B) and approximately 29 hours after the initial injury demonstrating progressive erythema and swelling (C).

Concerned for an injury to the extensor tendon or a retained foreign body, the patient underwent musculoskeletal ultrasound of the dorsal hand later that evening (Figure 2). Ultrasound demonstrated two distinct cortical interruptions along the dorsal surface of the third metacarpal head with moderate surrounding edema and prominent hyperemia surrounding the cortical break. The adjacent extensor tendon was intact. 

**Figure 2:**
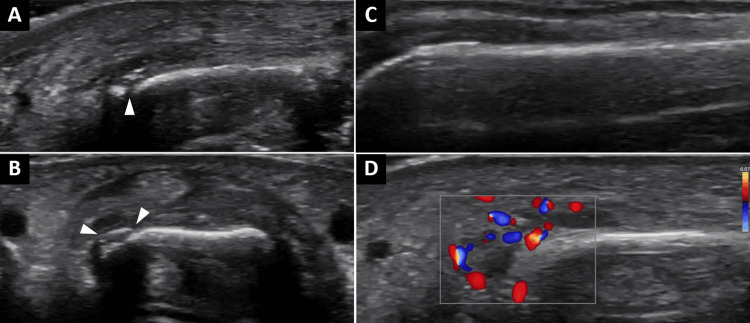
Diagnostic ultrasound of the third metacarpal head Long axis (A) and short axis (B) images of the head of the right third metacarpal demonstrate focal cortical disruptions (arrowheads). A comparison of the contralateral hand in long axis (C) demonstrates a smooth, uninterrupted cortex of the dorsal surface of the left third metacarpal. Color Doppler (D) demonstrates significant hyperemia surrounding the cortical disruption.

Two days later, the patient presented to an orthopedic hand specialist, where he reported a decrease in swelling, redness, and pain. Upon physical examination, the patient was unable to make a full fist or achieve active metacarpophalangeal extension beyond 180 degrees. A three-view radiographic study of the right hand was ordered (Figure 3), which demonstrated questionable punctate lucencies at the head of the third metacarpal. The initial impression based on these findings was for a small chip fracture of the head of the third metacarpal. The patient was instructed to rest the right hand with no specific limitations aside from avoiding painful activities.

**Figure 3:**
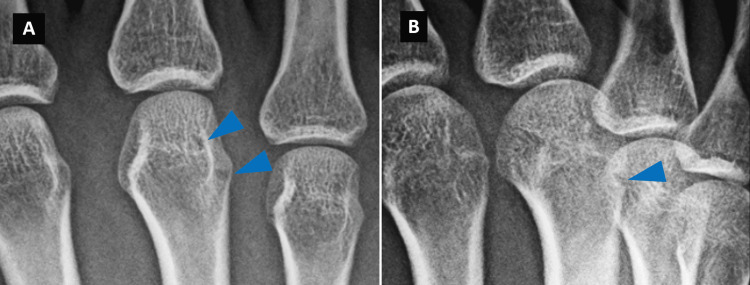
Radiographs of the right hand Frontal (A) and oblique (B) cropped views of the right hand demonstrating subtle cortical lucencies (arrowheads) along the dorsal/medial aspect of the third metacarpal cortex without evidence of a radiopaque foreign body.

One week after injury, pain, swelling, and erythema had substantially improved, and the patient was able to make a full fist. However, he remained unable to actively extend the metacarpophalangeal joints past neutral from a flat tabletop position. Persistent extension limitation nine days after the injury raised concern for a more complex occult injury. A follow-up ultrasound (Figure 4) was performed the following day and showed an enlarging cortical opening with findings suspicious for a retained foreign body.

**Figure 4:**
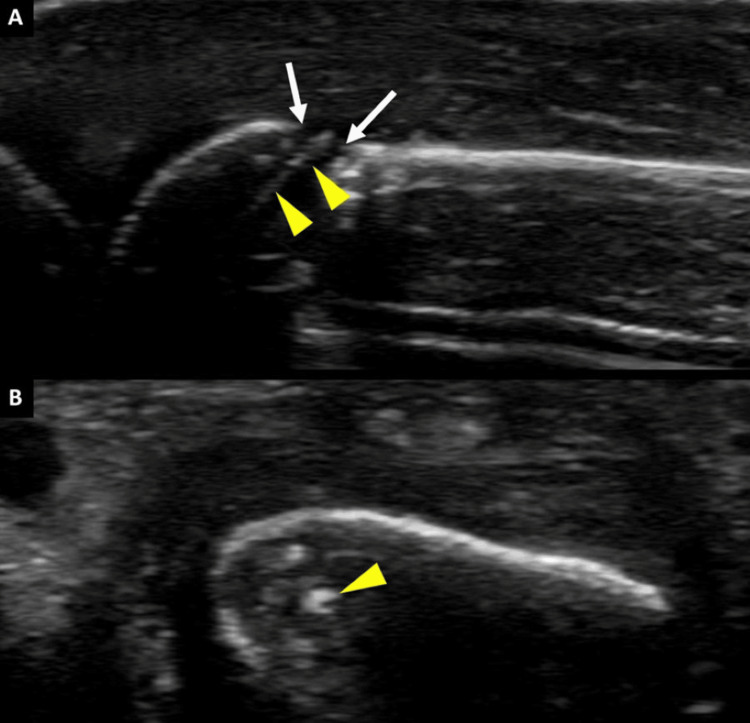
Follow-up diagnostic ultrasound Long axis (A) and short axis (B) follow-up diagnostic ultrasound images of the head of the third metacarpal demonstrate widening of the previously visualized cortical interruption (arrows) and a thin, linear intramedullary hyperechoic structure (arrowheads), suggestive of a retained foreign body.

MRI of the hand without contrast was obtained the same day (Figure 5). MRI demonstrated an enlarging, fluid-filled cavity within the medullary bone of the third metacarpal with adjacent cortical disruption, substantial surrounding bone marrow edema, and an internal linear signal void consistent with a retained foreign body. Early osteomyelitis was included in the differential diagnosis. The patient returned to the orthopedic surgeon the next day. Surgical debridement was recommended, with possible bone-filling cement depending on intraoperative findings.

**Figure 5:**
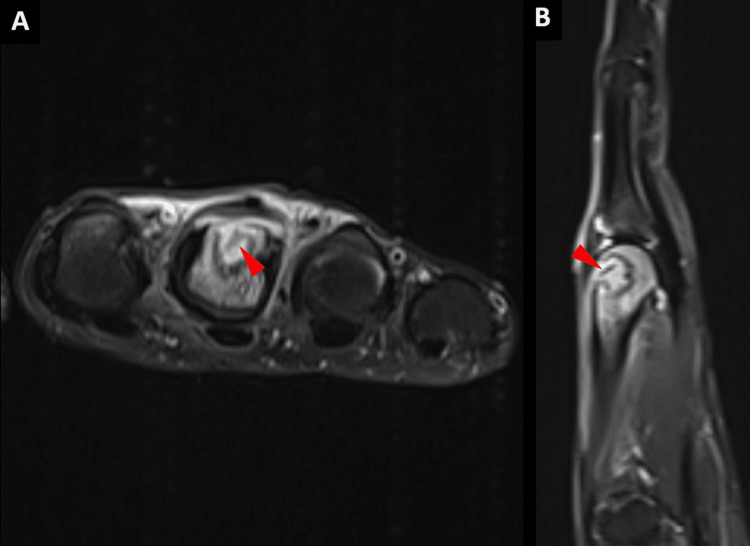
MRI of the right hand. Axial proton density fat-suppressed (A) and sagittal proton density fat-suppressed (B) images demonstrate a fluid-filled cavity within the head of the third metacarpal, containing a low signal, linear structure (arrowheads), suggestive of a retained foreign body. Significant reactive bone marrow edema surrounding the fluid-filled cavity and a cortical disruption of the dorsal cortex are also noted.

Surgery was performed approximately 12 days after the initial injury. Debridement was successful, bone cement was not required, and a 6 mm wooden fragment was extracted (Figure 6). Postoperatively, the patient was prescribed another course of oral antibiotics and pain medication, the hand was splinted, and right-hand lifting was restricted to less than three pounds.

**Figure 6:**
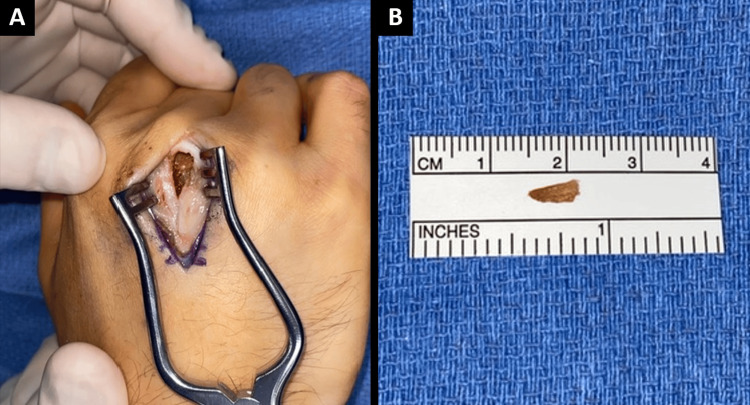
Surgical and specimen images. Image of the surgical procedure (A) demonstrating the cavity within the third metacarpal and an image of the removed foreign body (B) that measured approximately 6 mm.

The patient discontinued pain medication and returned to modified Pilates three days after surgery. Six days after surgery, the patient discontinued use of the splint and began rehabilitation, which amounted to only 18 days elapsed between the initial injury and physical therapy initiation (Figure 7). Symptoms continued to improve with physical therapy, and 16 weeks after surgery, the patient reported a nearly complete reduction in pain and a nearly full range of motion. He was able to return to playing disc golf competitively within six months of the initial injury.

**Figure 7:**
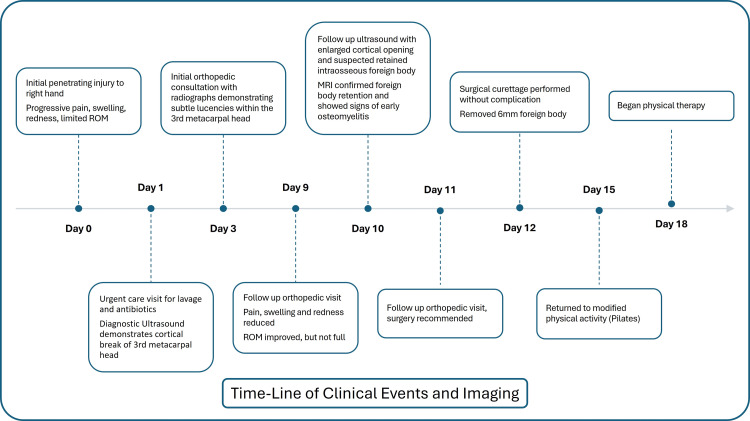
Timeline of clinical events and imaging ROM: range of motion

Patient perspective

After striking the tree branch, I felt significant pain and saw a piece of wood protruding from the back of my hand. It was firmly embedded, but I was able to pull it out myself, after which the wound bled heavily. I cleaned and dressed the wound and continued playing, but over the next several hours, I noticed increasing swelling, redness, and difficulty making a fist. Because I have type 1 diabetes and had experienced skin infections before, I was concerned when the redness continued to spread and sought urgent care.

Although antibiotics helped the pain and swelling, and redness improved, I remained unable to fully extend my middle finger. That persistent limitation made me seek further evaluation. After imaging and surgery, a 6-mm wooden fragment was removed from the bone. My recovery was uncomplicated overall, with temporary difficulty typing, and I returned to modified physical activity within a few days. This experience taught me to take penetrating hand injuries and persistent motion limitations seriously.

## Discussion

This case demonstrates several diagnostic pitfalls in penetrating hand trauma. The patient removed an obvious splinter immediately after injury and later underwent lavage and inspection, which lowered suspicion for retained material. However, wood fragments can break, fragment, and remain embedded despite wound care. Persistent swelling, spreading erythema, pain with motion, and functional limitation after a penetrating injury should maintain clinical suspicion for retained foreign body even if initial inspection and radiographs are unrevealing.

The radiographic findings in this case were subtle and equivocal, which is consistent with the known limitations of plain films for wooden material. In the classic 200-patient hand foreign body series by Anderson et al., wood, glass, and metal accounted for 95% of foreign bodies; wood was seen on only 15% of radiographs, whereas metal and glass were far more readily visible [10]. This supports the clinical lesson that a negative or minimally abnormal radiograph does not exclude retained wood after penetrating hand trauma.

Ultrasound was important in this case for two reasons. First, the initial study localized cortical disruption at the symptomatic dorsal third metacarpal head. Second, a repeat ultrasound demonstrated progression, showing an enlarging cortical opening and prompting further investigation. Ultrasound is particularly useful for foreign bodies because it can be performed dynamically, can evaluate superficial structures in multiple planes, and can localize foreign bodies relative to tendons, vessels, and bone [4]. Intra-osseous evaluation of foreign bodies is limited with ultrasound, as a cortical break is typically necessary for intramedullary visualization. This case demonstrates the potential value of diagnostic ultrasound for the evaluation of intra-osseous retained foreign bodies. 

MRI added value by demonstrating intramedullary involvement and a linear internal signal void consistent with retained wood. In a retrospective series of 18 surgically confirmed wooden splinter extremity injuries, MRI localized the splinter in 16 patients and described wooden fragments as linear signal voids on T1, T2, and proton density-weighted images surrounded by reactive inflammatory changes [11]. In the present case, MRI helped support operative planning and brought to light the concern for osteomyelitis.

Foreign body-associated osteomyelitis of the hand is uncommon but well described. A 2021 case report described a pediatric patient with hand osteomyelitis due to a missed plant thorn, highlighting how foreign body osteomyelitis can mimic other radiologic diagnoses [12]. A recent 2025 case report described chronic hand osteomyelitis due to retained wood for 12 years, underscoring the potential morbidity of delayed recognition [13]. Compared with chronic cases where diagnosis is delayed, the present case is notable for early recognition of a foreign body followed by surgical extraction within approximately two weeks of injury [13].

## Conclusions

Retained wooden foreign bodies should remain in the differential diagnosis after penetrating hand trauma, even when the patient removes an apparent splinter, and initial radiographs are negative or equivocal. Persistent erythema, swelling, pain, or functional limitation should prompt further imaging. Diagnostic ultrasound may assist in the identification of cortical, intramedullary, and soft-tissue abnormalities in the setting of penetrating trauma, leading to early recognition and improved outcomes. 
